# Supplementary material for: PDF-1 neuropeptide signaling regulates sexually dimorphic gene expression in shared sensory neurons of C. elegans
Source: eLife. 2018 Jul 19;7:e36547. doi: 10.7554/eLife.36547 (PMC6053303; doi:10.7554/eLife.36547)
Supplement: Supplementary file 1. — A comprehensive list of the strains used in this study. Strain source (this study or others) is indicated. [file elife-36547-supp1.pdf]

Supplementary File 1. *C. elegans* strains used in this study.

STRAIN NAME	GENOTYPE	SOURCE
CB1490	*him-5(e1490)*	Caenorhabditis Genetics Center
ZD1005	*ksIs2;him-5(e1490)*	Hilbert and Kim, 2017
ZD1309	*daf-7(ok3125); him-5(e1490)*	Hilbert and Kim, 2017
ZD1482	*him-5(e1490); ofEx4*	Hilbert and Kim, 2017
ZD1852	*ksIs2; pdfr-1(lst34); him-5(e1490)*	This paper
ZD1962	*ksIs2; pdf-1(tm1996); him-5(e1490)*	This paper
ZD1987	*ksIs2; pdfr-1(ok3425); him-5(e1490)*	This paper
ZD1997	*ksIs2; him-5(e1490); pdf-2(tm4393)*	This paper
ZD2033	*him-5(e1490); qdEx149[pdf-1(+) + pofm-1::GFP]*	This paper
ZD2076	*pdf-1(tm1996); him-5(e1490)*	This paper
ZD2084	*ksIs2; pdfr-1(ok3425); him-5(e1490); qdEx150[trx-1p::pdfr-1 cDNA (B isoform)::2A::mCherry +pofm-1::GFP]*	This paper
ZD2085	*ksIs2; pdfr-1(ok3425); him-5(e1490); qdEx151[wrm0629dH07(pdfr-1 genomic) +pofm-1::GFP]*	This paper
ZD2094	*pdfr-1(ok3425); him-5(e1490)*	This paper
ZD2106	*pdfr-1(ok3425); him-5(e1490); ofEx4*	This paper
ZD2138	*daf-7(ok3125); him-5(e1490); qdEx149*	This paper
ZD2264	*ksIs2; him-5(e1490); qdEx161[ptrx-1::acy-1(P260S)+pofm-1::gfp]*	This paper
ZD2266	*ksIs2; him-5(e1490); qdEx152[ptrx-1::acy-1(P260S)+pofm-1::gfp]*	This paper
ZD2269	*ksIs2; him-5(e1490); pdfr-1(ok3425); qdEx152*	This paper
ZD2278	*him-5(e1490); ofEx4; qdEx153[wrm0629dH07(pdfr-1 genomic)+pofm-1::gfp]*	This paper
ZD2281	*ksIs2; pdfr-1(ok3425); him-5(e1490); qdEx158[trx-1p::nCre+myo-2p::mCherry]*	This paper
ZD2282	*ksIs2; pdfr-1(ok3425); him-5(e1490); qdEx159[trx-1p::nCre+myo-2p::mCherry]*	This paper
ZD2290	*ksIs2; pdfr-1(ok3425); him-5(e1490); qdEx160[pSF11(tag-168p::Cre)+myo-2::mCherry]*	This paper
ZD2292	*ksIs2; pdfr-1(ok3425); him-5(e1490); qdEx157[pdfr-1p(5 kb distal)::loxP::pdfr-1B::loxP+ofm-1p::gfp]*	This paper
ZD2340	*pdf-1(tm1996); him-5(e1490); qdEx167[trx-1p::daf-7+ofm-1p::gfp]*	This paper
ZD2355	*ksIs2; him-5(e1490); qdEx165[trx-1p::pdfr-1(SAS)+myo-2p::mCherry]*	This paper

ZD2356	*ksIs2; him-5(e1490); qdEx166[trx-1p::pdfr-1(SAS)+myo-2p::mCherry]*	This paper
ZD2360	*ksIs2; him-5(e1490); qdEx163[trx-1p::gfp(SAS)+myo-2p::mCherry]*	This paper
ZD2362	*ksIs2; him-5(e1490); qdEx164[trx-1p::gfp(SAS)+myo-2p::mCherry]*	This paper
