# Supplementary material for: PDF-1 neuropeptide signaling regulates sexually dimorphic gene expression in shared sensory neurons of C. elegans
Source: eLife. 2018 Jul 19;7:e36547. doi: 10.7554/eLife.36547 (PMC6053303; doi:10.7554/eLife.36547)
Supplement: Supplementary file 2. — A comprehensive list of the oligos created for this study. All sequences are listed in the 5’ to 3’ direction. [file elife-36547-supp2.pdf]

Supplementary File 2. Oligos used for transgenic strain construction and *pdfr-1* FISH

All sequences are listed in the 5' to 3' direction.

**Primers used to generate *pdf-1* overexpression construct**	
CTTGGCGTAATCATGGTCATAG	pUC19 forward
TTCACTGGCCGTCGTTTTACAAC	pUC19 reverse
gtaaaacgacggccagtgaaAAAAGGTTGCTTTACCGAGAC	pdf-1 genomic forward w/pUC19 overlap
atgaccatgattacgccaagGCCACTTTTCACGCTTCAG	pdf-1 genomic reverse w/pUC19 overlap
**Primers used to generate ASJ-specific *pdfr-1* rescue construct**	
GTCCAATTACTCTTCAACATCC	pUC19+unc-54 3'UTR forward
TTCACTGGCCGTCGTTTTAC	pUC19+unc-54 3'UTR reverse
acgttgtaaaacgacggccagtgaaTGAGTTGGCACTTCGTAG	trx-1p forward w/ pUC19 overlap
tgatatgagtcagGATCAATTGCTCAAAGTCAC	trx-1p reverse w/pdfr-1 overlap
tgagcaattgatcCTGACTCATATCATGGCGG	pdfr-1 cDNA forward w/trx-1p overlap
ttaagggtctgcttgactggTGGAGATTTTGTGGAGCG	pdfr-1 cDNA reverse w/2A overlap
acccaccgtgtcaCCAGTCAAGCAGACCCTTAACTTC	F2A::mCherry forward w/pdfr-1 overlap
tagggatgttgaagagtaattggacCTTATACAATTCATCCATGCC	F2A::mCherry reverse w/unc-54 3'UTR overlap
**Primers used to generate ASJ-specific expression of ACY-1(P260S)**	
GTCCAATTACTCTTCAACATCC	trx-1p+unc-54 3'UTR backbone forward
GATCAATTGCTCAAAGTCAC	trx-1p+unc-54 3'UTR backbone reverse
gactttgagcaattgatcATGGACGACGATGTCGGTG	acy-1 forward w/ptrx-1 overlap
gttgaagagtaattggacTTAAAATTCGTCTTCTTGGTTATCCTTG	acy-1 reverse w/unc-54 3'UTR overlap
**Primers used to generate ASJ-specific RNAi constructs**	
acgttgtaaaacgacggccagtgaaTGAGTTGGCACTTCGTAG	trx-1p Forward w/pUC19 overlap
tccagtttccGATCAATTGCTCAAAGTCAC	trx-1p Reverse w/pdfr-1 sense overlap
tgtgtcaacaGATCAATTGCTCAAAGTCAC	trx-1p Reverse w/pdfr-1 anti-sense overlap
tccactgacaGATCAATTGCTCAAAGTCAC	trx-1p Reverse w/gfp sense overlap
ctgtgctcccGATCAATTGCTCAAAGTCAC	trx-1p Reverse w/gfp anti-sense overlap

gcaattgatcGGAAACTGGAATTCGTGG	pdfr-1 sense Forward w/trx-1 overlap
atgaccatgattacgccaagTGTTGACACATCCTGCTG	pdfr-1 sense reverse w/pUC19 overlap
gcaattgatcTGTTGACACATCCTGCTG	pdfr-1 antisense Forward w/trx-1 overlap
atgaccatgattacgccaagGGAAACTGGAATTCGTGG	pdfr-1 antisense Reverse w/pUC19 overlap
gcaattgatcTGTCAGTGGAGAGGGTGAAG	gfp sense Forward w/trx-1 overlap
atgaccatgattacgccaagGGGAGCACAGGGAGAAAG	gfp sense Reverse w/pUC19 overlap
gcaattgatcGGGAGCACAGGGAGAAAG	gfp antisense Forward w/trx-1 overlap
atgaccatgattacgccaagTGTCAGTGGAGAGGGTGAAG	gfp antisense Reverse w/pUC19 overlap
tgagttggcacttcgtaga	nested trx-1p Forward
TGCAAATTCTTACCACTGCATCT	nested pdfr-1 sense R
aaactatgactcggcgcaac	nested pdfr-1 antisense R
TTAGAAGTCAGAGGCACGGG	nested gfp sense R
TGCACTACTGGAAAACTACCTG	nested gfp antisense R
*pUC19 backbone was amplified with primers listed above and used for all Gibson cloning of RNAi constructs	
**Primers used to generate floxed *pdfr-1* rescue construct and ASJ-specific Cre**	
GGCGCGCCTCTAGAGGATC	pSF11 forward for amplifying Cre in plasmid backbone w/out promoter
GGCCGGCCCAGTCAGTGC	pSF11 reverse for amplifying Cre in plasmid backbone w/out promoter
ccgcactgactgggccggccTGAGTTGGCACTTCGTAG	trx-1p forward w/pSF11 overlap
ggatcctctagaggcgcgccGATCAATTGCTCAAAGTCAC	trx-1p reverse w/Cre overlap
ataacttcgtatagcatacattatacgaagttatGTCCAATTACTCTTCAACATCCC	pPD95.75 Forward w/loxP overlap
GTCGACCTGCAGGCATGC	pPD95.75 reverse primer
taagcttgcatgcctgcaggtcgacAAAAGGTTAAGCGCCCTTG	pdfr-1p(5kb) Forward w/pPD95.75 overlap
gtatgctatacgaagttatTTCAATTCGACGTTTGACC	pdfr-1p(5kb) Reverse w/loxP overlap
agaattggtcaaacgtcgaattgaaataacttcgtatagcatacattatacgaagttatCTGACTCATATCATGGCG	pdfr-1 cDNA Forward w/loxP site and pdfr-1p(5kb) overlap
tagggatgttgaagagtaattggacataacttcgtataatgtatgctatacgaagttatTTATGGAGATTTTGTGGAGC	pdfr-1 cDNA Reverse with loxP site and pPD95.75/unc-54 3'UTR overlap

***pdfr-1* FISH Probe Set**	
accataagcattgcaatcat	
caatatagagtaccagccga	
ccattttcaccagtgaatat	
ttctgctgagttcgtgtgag	
ggcatagtatttatcgtttt	
gaacattccttcacaaacga	
attccttctaagaacatcca	
agggtaacaattttggctcc	
ataccgtatccagcaatgaa	
tagccagagcattgtgtgaa	
ctttttgatgagcaccaca	
taagcatcgttccactttga	
ctaggaccatctagaatcca	
atttatcacaagttctgcca	
gcactcgaatgacattacag	
agattctcgaactttactgt	
agacctgcttcagacgtatt	
attgcagctttgaccgattt	
acctaacagcggcaataaca	
ttgtttgcattatattcgga	
ttatccctagttggtgcaaa	
cgtccaaacagcaaatacca	
tataggtgaaagaggcggtg	
atactggcaaccataagtcc	
aacctccttgttcgtaaagc	
agaaggtcttgaggacgtga	
ttatggaggagtcgataccg	
tcgtcgaagttcgttttgtg	
ataatgggatgcgacgcttc	
ttagctgttccattttttgc	
tatttgtctgtggtgcactt	
agttttccaaactcgtcagc	
ttttactccttgacgggaac	
cgtcgtggaatcatcgctac	
catcactgcatctttcatca	
tgtttgcattcttttcttct	
